# Convenient broad-host-range unstable vectors for studying stabilization cassettes in diverse bacteria

**DOI:** 10.1186/s12866-016-0674-y

**Published:** 2016-04-05

**Authors:** Aneta A. Bartosik, Krzysztof Glabski, Anna Kulinska, Ewa Lewicka, Jolanta Godziszewska, Aleksandra Markowska, Grazyna Jagura-Burdzy

**Affiliations:** Department of Microbial Biochemistry, Institute of Biochemistry and Biophysics, Polish Academy of Sciences, Pawinskiego 5A, 02-106 Warsaw, Poland; Present address: Faculty of Chemistry, Institute of Biotechnology, Warsaw University of Technology, Warsaw, Poland; Present address: Faculty of Human Nutrition and Consumer Sciences, Laboratory of Food Chemistry, Warsaw University of Life Sciences, Warsaw, Poland; Present address: Pulawska 255A/4, 02-740 Warsaw, Poland

**Keywords:** Broad-host-range, Cloning vector, RK2, *lacZ*, *xylE* reporter, Stability functions

## Abstract

**Background:**

Low-copy-number vectors of potential wide application in biotechnology need to encode stabilization modules ensuring their stable inheritance. The efficiency of stabilization may vary depending on the plasmid host so a thorough analysis of stabilization functions is required before use.

**Results:**

To facilitate such analysis highly unstable, mobilizable, broad-host-range (BHR) vectors based on RK2 replicon were constructed. The vectors are suitable for testing of various stabilization functions, including plasmid and chromosomal partitioning cassettes encoding ParB homologues capable of spreading on DNA. The *xylE* or *lacZ* reporter systems facilitate easy monitoring of plasmid segregation.

**Conclusion:**

The range of BHR vectors with different reporter cassettes and alternative mobilization systems expands their application in diverse bacterial species.

## Background

The stabilization functions carried by low-copy-number plasmids from a wide range of bacteria ensure their stable inheritance during cell division [1]. Putative stabilization modules (e.g., partitioning operons, toxin-antitoxin systems, restriction-modification mechanisms) are also encoded on bacterial chromosomes [2–6]. Such modules could be used to construct vectors for biotechnological applications. The properties of the stabilization modules may vary depending on the host they are expressed in, so a thorough analysis is required before use.

Several test vectors are available for studying stabilization functions in bacteria. Most of them rely on narrow-host-range replicons and can be used only in certain *E. coli* strains or other narrowly defined hosts [7, 8]. pALA136 [9] and pOG04 [10] are based on dual pMB1 and P1 or P7 replicons, respectively. The high-copy-number pMB1 replicon requires PolI for replication, so the plasmid is stable in *polA*^+^ strains but when transformed into a *polA* mutant, it depends on the phage vegetative replication system and consequently becomes highly unstable as a single-copy molecule unless a stabilization cassette is included.

The standard method for testing putative stabilization functions relies on a classical segregation test, in which a strain with the plasmid is cultured for a certain number of generations without selection and then the number of cells still carrying the plasmid is estimated by the time-consuming replica plating of colonies or serial dilutions (drop test). Introduction of the reporter gene *lacZ* in pOU82 [11] simplifies the screening for plasmid loss, but this very useful test vector can only be applied for *E. coli* and its closely related species since it relies on the narrow-host-range R1 replicon of the IncFII incompatibility group [12].

This paper presents a set of highly unstable broad-host-range plasmids based on the RK2 replicon of IncP-1 group [13] designed to test stabilization functions in diverse bacterial species. pABB35 and its derivatives are single-copy vectors specifying chloramphenicol resistance (Cm^R^). The multiple cloning site (MCS) is flanked by *lacO* operators serving as binding sites for LacI repressor to build a roadblock for polymerizing ParB-type proteins encoded by the tested partitioning cassettes of type IA [14, 15]. The unstable vectors contain the *xylE* (*klcA*p_RA3_*-xylE-T*_*pro/lyz*P1_) or *lacZ* (*klcA*p_RA3_*-lacZ-T*_*pro/lyz*P1_) reporter gene enabling easy and quick detection of bacterial colonies retaining the plasmid with the potential stabilization cassette. The plasmid segregation process can also be monitored in liquid cultures by a quantitative XylE activity assay. Variants of the unstable vector mobilizable by the RK2 (IncP-1) conjugative system integrated into the *E. coli* chromosome or by the RA3 (IncU) one integrated into the *Pseudomonas putida* chromosome are also available.

## Results and discussion

### Construction of a highly unstable broad-host-range plasmid

The main aim of this project was to engineer an unstable cloning vector suitable for easy monitoring of segregation functions in a wide range of bacteria.

We chose pRK415 [16], a derivative of the RK2 replicon from the IncP-1α incompatibility group, to construct a highly unstable broad-host-range (BHR) test vector.

The pRK415 cloning vector contains four following RK2 fragments: i/ a region encoding Ssb (single-stranded DNA-binding protein), the replication initiator protein TrfA, Upf16.5 of unknown function [13], and TrbA, a regulatory protein of RK2 conjugative transfer operons [17]; ii/ *oriV*_RK2_ with eight iterons constituting TrfA binding sites [18, 19]; iii/ part of the central control operon *korA-incC* encoding KorA, the primary repressor of *trfA*p [20], since strong *trfA*p is unclonable when unregulated [21], and iv/ the *traJ traK* intergenic region with *oriT*_RK2_ to facilitate mobilization by the RK2 conjugation system [22]. Additionally, the vector carries a tetracycline resistance cassette (Tc^R^) and a *lac*p-*lacZ* fragment with MCS for α-complementation and easy identification of cloned inserts. pRK415 had previously been reported as slightly unstable [16, 23], but our plasmid retention tests showed its almost 100 % stability, since after 40 generations of growth of *E. coli* DH5α (pRK415) under non-selective conditions in L broth without antibiotics almost 100 % of cells retained the plasmid (Fig. 2a).

**Fig. 1:**
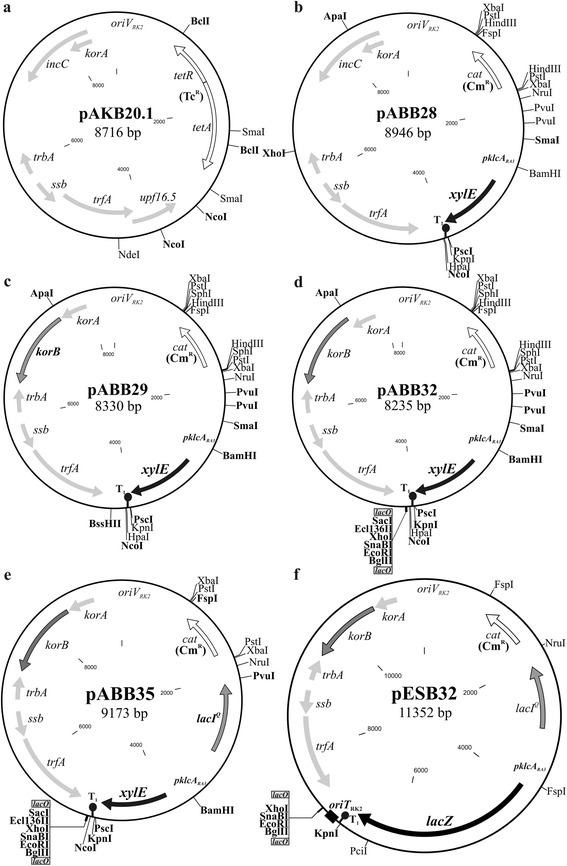
Milestones in construction of unstable BHR vector. Circular maps of intermediate (**a, b** and **c**) and final vectors (**d, e** and **f**) are drawn to scale. Only intact orfs are indicated. Unique or double restriction sites important for cloning are shown, those described in the text are in bold. T_1_ marks the divergent transcription terminator sequence T_*pro*_/T_*lyz*_ of P1 prophage [49]

The strategy to obtain a truly unstable derivative of the RK2 minireplicon was to limit the expression of *trfA*, first by introducing a promoter-down mutation in *trfA*p and, if required, by adding KorB, a second repressor acting cooperatively with KorA on *trfA*p [24], to the system. It has previously been shown that the T → C mutation in the -10 sequence of *trfA*p (*trfA*p-1) decreases the promoter activity at least 10-fold [17]. Site-directed PCR mutagenesis was used to introduce *trfA*p-1 mutation together with an AatII restriction site into pRK415. Plasmid DNA sequencing confirmed the introduction of the desired mutation into the -10 sequence of *trfA*p, but also an unexpected deletion of 1974 bp encompassing *lacZ*α with MCS and the *traJ*-*traK* region with *oriT*_RK2_. The obtained 8716-bp derivative pAKB20.1 (Fig. 1a) was still very stably maintained in *E. coli* DH5α cells demonstrating 100 % retention after 40 generations of growth under non-selective conditions (data not shown).

**Fig. 2:**
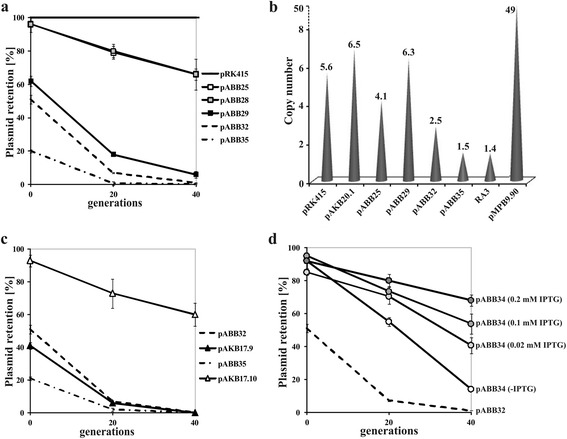
Standard stability tests of constructed vectors. *E. coli* DH5α transformants were grown overnight with antibiotic (point 0) and for 40 generations without antibiotic. Every 20 generations appropriate dilution was plated on L agar to obtain approximately 100 colonies. The colonies were replica plated to test for chloramphenicol resistance. Plasmid retention was expressed as percentage of Cm^R^ colonies. The results shown are average from three experiments with standard deviation. **a** Retention of constructed vectors. **b** Plasmid copy number estimated by RealTime qPCR. Plasmid copy relative to the chromosome was assayed in three independent biological samples with three technical replicates each. Average results for plasmids are presented with SD as follows: 0.06; 1.75; 0.21; 0.16; 0.95; 0.32; 0.17; 6.96, respectively. **c** Stabilizing properties of active partitioning operon from RA3 in pABB32 and pABB35 vectors. **d** Effect of IPTG-induced expression of *P. aeruginosa parA-parB* operon on pABB34 plasmid retention. DH5α(pABB34) cultures were grown in L broth with various concentrations of IPTG

To facilitate insertion of potentially large DNA fragments bearing stability cassettes it was required to downsize the cloning vector. Hence, pAKB20.1 was modified by NcoI digestion and self-ligation to delete a 527-bp fragment of the *upf16.5* gene of unknown function [13], the last orf in the *ssb-trfA-upf16.5* operon present only in one subgroup of IncP-1 plasmids (IncP-1α). Although an Upf16.5 function in copy number control (e.g., efficient replication initiation) has not been reported yet, the new derivative, pABB25, was slightly less stable in *E. coli* DH5α cells in comparison to pAKB20.1 and after 20 generations of growth without selection 20 % of cells lost the pABB25 plasmid (Fig. 2a). In the next step the tetracycline resistance operon (Tc^R^) of pABB25 was replaced with a shorter *cat* gene (encoding chloramphenicol acetyltransferase) conferring the Cm^R^ phenotype. The resulting plasmid pABB25.1 (Cm^R^) demonstrated stability in *E. coli* DH5α comparable to that of pABB25 (data not shown).

The initial manipulations did not sufficiently decrease the stability of the RK2 minireplicon, so it was decided to proceed with the addition of *korB* encoding a co-repressor of *trfA*p, to the system. It was also important to inactivate *incC* since IncC and KorB constitute the active partitioning system of RK2 [25, 26]. Two restriction sites, ApaI and XhoI were introduced into pABB25 to facilitate substitution of *incC2* orf with *korB*_RK2_ and to give pABB27. Before *korB* cloning, a SmaI-NcoI fragment encoding *klcA*p_RA3_-*xylE-*T_*pro/lyz*P1_ was inserted into pABB27 between the *trfA* and *cat* genes to give pABB28 (Fig. 1b). *klcA*p_RA3_ is a strong promoter and *xylE* encodes catechol 2,3- dioxygenase, whose activity is easy to be monitored following bacteria growth on plates [27] or in liquid cultures (this work). Subsequently, the *korB*_RK2_ was inserted into pABB28 between the XhoI and ApaI sites. The obtained pABB29 plasmid (Fig. 1c) demonstrated a high loss rate in *E. coli* DH5α strain (Fig. 2a). pABB29, comprising a modified RK2 replication system, the *korA-korB* operon, the *klcA*p*-xylE-T*_*pro/lyz*P1_ reporter cassette and the Cm resistance marker, was used in the next step to prepare the final version of the unstable BHR vector for cloning and testing stabilization cassettes in a wide range of bacterial species.

The MCS introduced between the unique NcoI and BssHII (PauI) sites in pABB29 to give pABB32 (Fig. 1d) contained BglII, EcoRI, SnaBI, XhoI, Ecl136 and SacI restriction sites and was surrounded by *lacO* operator sequences. The binding of LacI repressor to the *lacO* operators was expected to act as a roadblock [28] for potentially polymerizing ParB partitioning proteins encoded by type IA partition cassettes [14, 15] that might be analyzed using this vector. ParB spreading that follows its binding to *parS* (centromere-like sequence) may lead to transcriptional silencing of nearby genes and therefore affect results of segregation studies [29–33].

Finally, the *lacI*^*q*^ allele [34, 35] was inserted into pABB32 to obtain the final construct, the pABB35 vector (Fig. 1e). The *lacI*^q^ mutation refers to a change in the −35 motif of *lacI*p causing overexpression of *lacI* (superrepressor) [36] and is often used in recombinant strains or vectors to provide tighter control of *lacZ*p (or hybrid *tac*p) expression in the absence of the IPTG inducer.

The high instability of pABB35 was confirmed by the standard stability test: only approximately 2 % of cells retained the plasmid after 20 generations of growth without selection (Fig. 2a).

### Plasmid copy number

The copy number of chosen plasmid constructs described above was determined in *E. coli* cells by qPCR [37]. The pRK415 derivatives pAKB20.1, pABB25 and pABB29 were present in 4 to 6.5 copies per chromosome, similarly to pRK415 itself (Fig. 2b). The number of pABB32 and pABB35 copies was 1.5 - 2.5 per *E. coli* chromosome. This lower copy number is due to the tight regulation of *trfA*p-1 and underlies the instability of these test vectors. The copy number of pMPB9.90 *araBAD*p*-trfA*, based on pBAD24 [38], was established at 50 copies per chromosome in correlation with a published data [39]. For comparison also a single-copy-number plasmid RA3 of IncU group [40] was used and demonstrated 1–2 copies per chromosome (Fig. 2b).

### Plasmidic and chromosomal partitioning cassettes stabilize test plasmids in *E. coli*

The type IA active-partitioning cassette *korA-incC-korB-orf11-parS* from RA3 plasmid [40, 41] was chosen to check the applicability of the constructed vectors in a stabilization assay in bacteria. The cassette was cloned into pABB32 and pABB35 vectors to obtain pAKB17.9 and pAKB17.10, respectively, and both plasmids were tested in the standard stabilization assay in *E. coli* DH5α strain.

The RA3 partitioning cassette did not drastically improve the pABB32 plasmid segregation rate: approximately 10 % of cells retained the pAKB17.9 plasmid after 20 generations of growth without selection (Fig. 2c). Remarkably, the same RA3 fragment cloned into pABB35 exhibited the expected stabilization function and pAKB17.10 was retained in approximately 70 and 60 % of *E. coli* DH5α cells after 20 and 40 generations of growth without selection, respectively (Fig. 2c). The only difference between pABB32 and pABB35 is the presence of the *lacI*^*q*^ allele in the latter (Fig. 1d and e). Since it has been shown previously that KorB_RA3_ (a ParB homolog) spreads on DNA after binding to *parS* and silences nearby genes [41], the different stability of pAKB17.9 and pAKB17.10 convincingly demonstrates that overproduction of LacI and its binding to *lacO* sequences flanking the cloned stabilization cassette blocks effectively the KorB spreading.

The usefulness of the constructed vectors was also checked with a synthetic chromosomal partition cassette *lacI*^*q*^*-tac*p*-parA-parB-parS* from *P. aeruginosa* [29] cloned into pABB32 to give pABB34.

The pABB34 plasmid was present in more than 50 % of *E. coli* cells after 20 generations of growth without selection, in comparison to only 10 % of cells retaining empty pABB32 (Fig. 2d). After 40 generations pABB34 was still present in 14 % of cells, whereas the retention of pABB32 dropped below 1 %.

The stabilization effect of the RA3 partitioning cassette cloned in pAKB17.10 was stronger than that demonstrated by the *parA-parB-parS*_*P.a*_ cassette present in pABB34 (Fig. 2c and d). These differences in the stabilization potential could reflect the individual properties of each cassette, but the rather modest effect of the synthetic *parA-parB-parS* cassette of *P. aeruginosa* could also be due to the low amount of partitioning proteins produced since the *parA-parB* operon in pABB34 is expressed at a low basal level from the strongly repressed *tac*p. To check which explanation was correct, different concentrations of IPTG were used to boost the production of the partition proteins ParA and ParB. In support of the latter possibility IPTG at 0.02-0.2 mM improved the stability of pABB34 (Fig. 2d). A further increase in IPTG concentration (0.5 mM) did not improve the plasmid stability (data not shown) probably due to the antagonistic effect of IPTG on the action of LacI as the roadblock to ParB, known to spread on DNA starting from *parS* [29].

### Catechol 2,3-dioxygenase activity assay as an estimate of plasmid stability

The results presented in Fig. 2 come from a standard stabilization assay with the use of replica plating to estimate the proportion of colonies retaining the plasmid tested (in this case, conferring resistance to chloramphenicol). The *xylE* reporter cassette present in pABB32 and pABB35 allows the plasmid segregation to be assayed using a simple plate test to visualize colonies that express the *xylE* reporter gene and hence must have retained the plasmid.

**Fig. 3:**
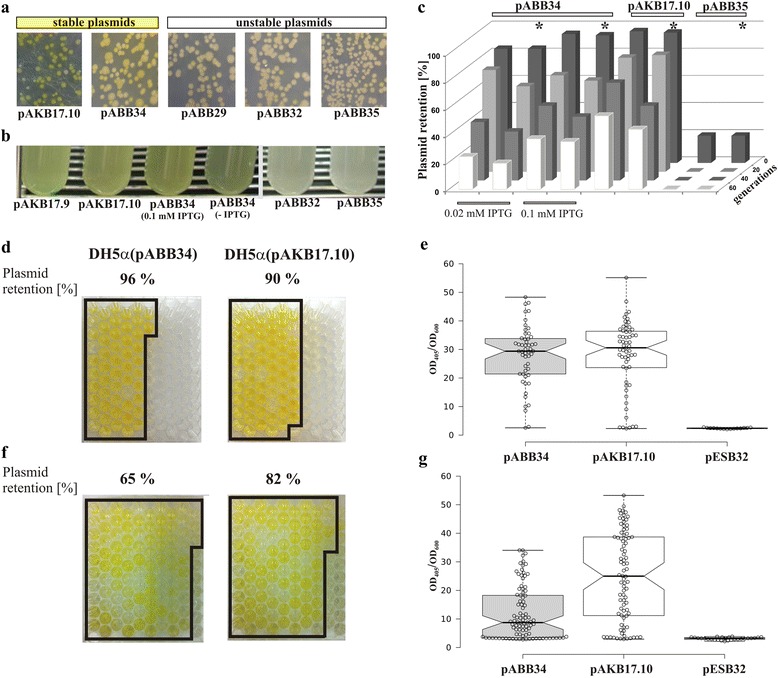
Detection of plasmid retention on plates, in liquid cultures and by high-throughput quantification. **a** Transformants of *E. coli* DH5α with stable (pAKB17.10 or pABB34) and unstable (pABB29, pABB32 or pABB35) plasmids were grown for 20 generations without selection and IPTG and then 100 μl of 10^6^-fold dilutions was plated on L agar, sprayed with 10 mM catechol and photographed. **b** Overnight cultures of *E. coli* DH5α transformants bearing indicated plasmids were grown for 20 generations in L broth and 0.1 mM IPTG where marked. Catechol was added to 1 mM to the cultures and after 5 min of incubation tubes were photographed. **c** Comparison of segregation data obtained in the standard replica plating test and visualization test (marked by asterisk) with the use of catechol as in (**a**). The results shown are representative of three independent experiments. **d/f** High-throughput quantification of plasmid retention in single-cell subcultures. Overnight cultures of DH5α(pAKB17.10) and DH5α(pABB34) grown in L broth with antibiotic (no IPTG added) were diluted to 5 cells ml^−1^ in L broth and aliquoted into 100-well plates (200 μl/well). After growth in Bioscreen (ca. 20 generations) the subcultures were diluted 100-fold into a new plate and 1/10 vol of 10 mM catechol was added to each well and photographed (**d**). Similar tests (**f**) were carried out after 40 generations of growth without selection (24 h in tubes followed by 24 h in Bioscreen plate). The photographs were taken after 10 min of incubation with catechol. The plasmid retention in liquid cultures (initial and after 20 generations of growth without selection in tubes) corresponds to the percentage of single-cell subcultures turning yellow. **e/g** The colour development quantified by OD_405_ after addition of catechol to the single-cell subcultures in the wells from (**d**) and (**f**), respectively. DH5α(pESB32) strain was used as a negative control. Plasmid segregation during growth without selection in the wells for 20 generations (**e**) and for total 40 generations (**g**) was reflected by variable level of XylE activity (OD_405_). OD_405_/OD_600_ ratio for each culture was plotted using BoxPlotR (boxplot.tyerslab.com; [55]). Boxes indicate the 25th and 75th percentiles and center lines show the medians. Whiskers mark minimum and maximum values in accordance with Spear criteria, and non-overlapping notches indicate that population medians are different with 95 % confidence as determined by R software

The cultures of transformants were grown without selection for a certain number of generations, diluted and plated onto L agar without antibiotics to get 100 to 200 colonies. The colonies were sprayed with 10 mM catechol and those derived from cells that had lost the test plasmid with *xylE* remained opalescent, those in which the test plasmid was stably maintained turned yellow quickly (Fig. 3a), whereas colonies of strains carrying an unstable plasmid with the *xylE* gene, were in various shades of yellow.

A quick semi-quantitative test for plasmid stability can also be performed for liquid cultures directly. Addition of catechol to overnight cultures (2x10^9^ cells ml^−1^) to 1 mM final concentration clearly distinguishes those in which XylE is produced by the majority of cells (the test plasmid is stably maintained) from the ones where the plasmid is hardly retained (high plasmid loss rate) (Fig. 3b). Care must be taken to determine the initial rate of reaction, i.e., to measure OD_405_ within a few minutes (2–5) after substrate addition [27, 34, 42].

### Comparison of the standard plasmid stability assay with plate catechol 2,3-dioxygenase determination

*E. coli* DH5α transformants bearing appropriate test plasmids were cultivated in L broth without antibiotics as earlier and tested for plasmid retention after approximately 20, 40 and 60 generations using, in parallel, the standard stabilization assay and the *xylE* plate test described above. As shown in Fig. 3c the results of plasmid retention estimation are quite similar for the two assays, justifying the use of the quicker *xylE* test.

### High-throughput analysis of plasmid stabilization functions

Plasmid retention was quantified in liquid cultures of DH5α(pAKB17.10) and DH5α(pABB34) using a high-throughput procedure. In this experiment the production of ParA and ParB in DH5α(pABB34) was not induced by IPTG to have two plasmids (pABB34 and pAKB17.10) stabilized to different extent by the various partition cassettes [compare Fig. 2c and graph marked pABB34 (no IPTG) in Fig. 2d]. DH5α(pABB35) and DH5α(pESB32) strains were used as controls. Cultures of the transformants were grown overnight under selection (10 μg ml^−1^ chloramphenicol) and then diluted to 5 cells ml^−1^ in L broth and aliquoted into 100-well plates (200 μl per well). The plates were incubated at 37 °C with shaking for ca. 20 generations. In parallel the overnight start cultures were diluted 10^5^-fold and grown in tubes for 20 generations without selection and then diluted, aliquoted and incubated as above (a total of 40 generations without selection). Overnight subcultures derived from single cells and grown in the 100-well plates were diluted 100-fold, OD_600_ values were measured and after addition of 10 mM catechol to each well (final concentration 1 mM) OD_405_ was read after 10 min. The results obtained after 20 generations for DH5α(pABB35) showed an almost complete lack of the plasmid indicating its high instability (only ca. 1.5 % of wells showed OD_405_ above background values obtained for the strain bearing pESB32 without *xylE*, data not shown). In the case of DH5α(pABB34) and DH5α(pAKB17.10) XylE activity was clearly detectable in 96 and 90 % of wells, respectively, after 20 generations of growth without antibiotic (Fig. 3d). After 40 generations of growth without antibiotic the corresponding values were 65 and 82 % for DH5α(pABB34) and DH5α(pAKB17.10), respectively (Fig. 3f). These results reflected well the differences in stability of the two plasmids observed in the standard and colony visualization assays when DH5α(pABB34) was grown without IPTG (Fig. 2d).

To normalize obtained data the OD_405_/OD_600_ ratio was calculated. It was around 2–3 for the control DH5α(pESB32) strain and varied between 2 and 50 for the DH5α(pABB34) and DH5α(pAKB17.10) strains (Fig. 3e and g). When the ratios were plotted and analyzed, the medians reflecting plasmid retention rates in the subcultures were similar for the two plasmids following growth without selection for 20 generations, and substantially higher for DH5α(pAKB17.10) compared with DH5α(pABB34) after 40 generations of growth without selection, as observed before.

The high-throughput approach is obviously more reliable than the standard and colony visualization method since human error is minimized and such a quantitative procedure may help to demonstrate even small, but statistically significant differences in stabilities between various plasmids in a given host, the same plasmid in various hosts, or between variants of the same plasmid.

### Modifications of the test vectors to expand their applicability

The test plasmid pABB35 based on the RK2 minireplicon can propagate in a variety of species. Since many bacterial species are not easily transformable, two different *oriT* regions amplified from BHR conjugative plasmids, RK2 and RA3, were inserted additionally to pABB35 to give pESB30 and pESB31, respectively. Such vectors are mobilizable during conjugation of the recipient with two different bacterial species, *E. coli* S17-1 with integrated RK2 plasmid [43] or *P. putida* KT2440 with integrated conjugation module of RA3 (KT2440 *tra*_RA3_), which may significantly extend the range of recipient strains.

The suitability of pESB30 vector for investigating stability mechanisms other than active partition was in the meantime confirmed by D. Bartosik’s group studying plasmidic toxin-antidote (TA) systems. The use of pESB30 vector with cloned *hipAB* system (TA) of pKON1 from *Paracoccus kondratievae* [44] allowed analysis of its stabilization functions in various species of Alphaproteobacteria e.g., *P. pantotrophus* and *Ochrobactrum sp.* (Czarnecki and Bartosik, personal communication).

Quick identification of colonies carrying the constructed vectors with the use of the color reaction enabled by *xylE* cassette could not be applied to some analyzed species e.g., *P. aeruginosa*, since when PAO1161 was transformed with *xylE* plasmids it formed yellow colonies due to the intrinsic substrates for catechol 2,3-dioxygenase. An alternative test vector, pESB32 (Fig. 1f), was constructed with the *klcA*p-*lacZ* cassette enabling monitoring of plasmid presence by formation of blue colonies in the presence of X-gal.

**Fig. 4:**
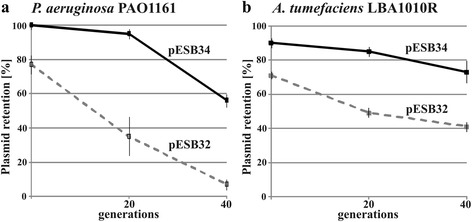
Functionality of test vectors in various bacterial species. **a** *P. aeruginosa* PAO1161 was transformed with pESB32 and pESB34 (*korA-incC-korB-orf11-parS*). Three transformants were grown for 40 generations without selection. The retention of plasmid was estimated by percentage of blue colonies on L agar plates with X-gal. The results shown are average from three experiments with standard deviation. **b** *A. tumefaciens* LBA1010R Rif^R^ was conjugated with *E. coli* S17-1(pESB32) and S17-1(pESB34) donor strains. Obtained transconjugants were selected on L agar supplemented with chloramphenicol and rifampicin. Three independent transconjugants were grown for 40 generations without selection. The retention of plasmid was estimated by percentage of blue colonies on L agar plates with X-gal. The results shown are average from three experiments with standard deviation

The partition operon *korA-incC-korB-orf11-parS* of RA3 was inserted between the EcoRI and XhoI sites in pESB32 to give pESB34. *P. aeruginosa* PAO1161 was transformed with pESB32 or pESB34 (*korA-incC-korB-orf11-parS*) and transformants were used to estimate plasmid retention (Fig. 4a). pESB32 or pESB34 were also used to transform *E. coli* S17-1 and transformants were applied as donors in conjugation with a Rif^R^ derivative of *A. tumefaciens* LBA1010R. The transconjugants were grown under selective conditions, then diluted appropriately and plated on L agar with X-gal. The retention rates of both plasmids assessed by the number of blue colonies are shown in Fig. 4b. pESB32 was less unstable in the both strains tested in comparison with the original pABB35 in *E. coli* DH5α (Fig. 2a) probably due to variations in the functioning of the copy-number control circuit of the RK2 minireplicon. The presence of the RA3 partition cassette still significantly increased the pESB34 retention in *P. aeruginosa* PAO1161 strain but had a much lower impact on plasmid stability in *A. tumefaciens* LBA1010R. The reasons for the observed differences in empty vector stability and the stabilization effects of a given cassette in various bacterial species await elucidation.

## Conclusions

We have manipulated the broad-host-range RK2 minireplicon pRK415 to obtain a highly unstable pABB35 vector. To facilitate easy monitoring of plasmid retention, a *xylE* or *lacZ* reporter system was inserted. A multiple cloning site surrounded by roadblocks for protein spreading enables analysis of various partition cassettes. To broaden applications of the vector to a variety of hosts we added *oriT* regions from RK2 or RA3 BHR plasmids so they could be mobilized by the widely used S17-1 (with RK2 integrated) and *P. putida* KT2440 with the *tra* module of RA3 constructed in this work, respectively.

The constructed vectors were demonstrated to be useful for cloning chromosomal and plasmid partition operons that produce type IA ParB-like proteins able to spread on DNA. Retention of the vectors varied depending on the host so they need to be tested in a given strain prior to application. Analysis of the stabilization properties of the cloned partitioning operons in three different hosts (*E. coli*, *P. aeruginosa*, *A. tumefaciens*) confirmed their variability and at the same time necessity to conduct such experiments.

Estimation of plasmid stability using catechol 2,3-dioxygenase assay in liquid cultures facilitates large scale or high-throughput experiments in bacteria, since hundreds or even thousands of variants can be monitored easily. It can be used to screen for new stabilization cassettes in meta-genomic approaches, to study stability functions in diverse bacteria and to screen mutant proteins affecting plasmid stability as well as inhibitors of the systems.

The test plasmids described here with an easy detection/monitoring system should be useful for studies of various stabilization functions in a wide range of strains in which the RK2 replicon can propagate.

## Methods

### Bacterial strains and growth conditions

*Escherichia coli* strains used were DH5α [F^*-*^(*Φ80dlacZ recA1 endA1 gyrA96 thi-1 hsdR17*(*r*_*k*_^*−*^*m*_*k*_^*+*^) *supE44 relA1 deoR* Δ(*lacZYA-argF*)*U196*] [45] and S17-1 [*recA pro hsdR* RP4-2-Tc::Mu-Km::Tn7] [43]. DH5α Rif^R^ mutant was selected during growth on L agar with 150 μg ml^−1^ rifampicin. *P. putida* KT2440 was kindly provided by C.M. Thomas (Birmingham University, Birmingham, United Kingdom). *P. putida* KT2440 *tra*_RA3_*korC* Km^R^ was constructed by integration of pJSB1.28 into a non-coding region of chromosome using homologous recombination (see below). *P. aeruginosa* strain PAO1161 (*leu*^*−*^*, r*^*−*^*, m*^*+*^) was kindly provided by B.M. Holloway (Monash University, Clayton, Victoria, Australia). *Agrobacterium tumefaciens* LBA1010R Rif^R^ was kindly provided by D. Bartosik (University of Warsaw, Warsaw, Poland). Bacteria were generally grown in L broth [46] at 37 °C or 28 °C (*A. tumefaciens*). L broth and L agar (L broth with 1.5 % w/v agar) were supplemented with appropriate antibiotics: chloramphenicol (10 μg ml^−1^ for *E. coli*, 50 μg ml^−1^ for *A. tumefaciens* and 150 μg ml^−1^ for *P. aeruginosa*), kanamycin (50 μg ml^−1^ for *E. coli* and *P. putida*) or tetracycline (10 μg ml^−1^ for *E. coli*). Strains with plasmids carrying the *klcA*p*-lacZ* fusion were tested on L agar with 40 μg ml^−1^ 5-bromo-4-chloro-3-indolyl-β-D-galactopyranoside (X-gal).

### Plasmid analysis and PCR amplification

Plasmid manipulations were carried out by standard procedures [47]. All plasmids constructed in this work are listed in Table 1.

**Table 1:** Plasmids used in this study

Plasmids provided by others or reported earlier:		
Name	Relevant features	References
pABB19	*oriV*_MB1_, Ap^R^, derivative of cloning vector pUC19 with added transcription termination sequence T_*pro*_/T_*lyz*_ from P1	[49]
pABB705	pKRP10 derivative with inactivated NcoI and PvuII sites in Cm^R^ cassette	[49]
pALA136	*oriV*_MB1_, *oriV*_P1_, Cm^R^_,_ dual replicon	[9]
pBGS18	*oriV*_MB1_, Km^R^, cloning vector	[56]
pBAD24	*oriV*_MB1_, Ap^R^, *araC*, *araBAD*p, expression vector	[38]
pCM132	*oriV*_ColE1_, *oriV*_RK2_, *oriT*_RK2_, *traJ’*, *trfA*, Km^R^, promoter-less *lacZ*, dual replicon	[52]
pGBT30	*oriV*_MB1_, Ap^R^, *lacI* ^*q*^ *, tac*p expression vector	[34]
pGEM-T-Easy	*oriV*_MB1_, Ap^R^, cloning vector	Promega
pJSB1.24	pBGS18 *tra*_RA3_ *korC*_RA3_ (RA3 coordinates 9437- 33857; 3093-3705)	[49]
pKLB3	pGBT30 *tac*p*-parA parB*_*P.a*_	[29]
pKRP10	*oriV*_MB1_, Ap^R^, Cm^R^	[50]
pMPB9.90	pBAD42 *araBAD*p-*trfA*_RK2_	Przyluski M.
pPT01	*oriV*_pSC101_, Km^R^, promoter-less *xylE*	[10]
pRK415	*oriV*_RK2_, Tc^R^, *oriT*_RK2_, stable vector	[16]
pYC16A	pALA136 with RA3 stabilization region	[41]
RA3	IncU, Cm^R^, Sm^R^, Su^R^	[40]
RK2	IncP-1α, Km^R^, Ap^R^, Tc^R^	Thomas C.M.
Plasmids constructed during this work:		
	Description, relevant features	
pABB18.1	pPT01 *klcA*p *-xylE,* PCR fragment amplified with primers #1 and #2 on RA3 template inserted as SphI-BamHI fragment	
pABB18.2	pABB18.1 cleaved with HpaI and NcoI, filled in and self-ligated to remove 561 bp upstream of *xylE*	
pABB18.3	pABB19 with *klcA*p*-xylE* inserted as SmaI-PscI PCR fragment amplified with primers #3 and #4 on pABB18.2 template	
pABB18.4	pBGS18 with *korB*_RK2_ inserted as EcoRI-SalI PCR fragment amplified with primers #5 and #6 on RK2 template	
pABB18.5	pGEM-T-Easy with *lacI* ^*q*^ gene PCR-amplified primers #11 and #12 on pGBT30 template	
pABB25	pAKB20.1 with 527-bp NcoI fragment removed	
pABB25.1	pABB25 with *cat* gene (Cm^R^) on BamHI fragment from pABB705 replacing BclI fragment from Tc^R^ cassette	
pABB26	pABB25.1 with XhoI restriction site introduced downstream of *trbA* (PCR directed mutagenesis with primers #20 and #21)	
pABB27	pABB26 with ApaI restriction site downstream of *korA* gene (PCR directed mutagenesis with primers #22 and #23)	
pABB28	pABB27 with *klc*p*-xylE* cassette, SmaI-NcoI fragment from pABB18.3	
pABB29	pABB28 with *korB*_RK2_ gene, ApaI-SalI fragment from pABB18.4 inserted between ApaI and XhoI sites	
**pABB32**	pABB29 with MCS flanked with *lacO* operators inserted between BssHII and NcoI sites, unstable vector	
pABB33	pABB32 with *lacI* ^*q*^ *-tac*p*-parA-parB*_*P.a.*_, DraI-SalI fragment of pKLB3 inserted between SnaBI and XhoI sites	
pABB34	pABB33 with *parS*_*P.a*._, annealed oligonucleotides #9 and #10 inserted into BglII restriction site	
**pABB35**	pABB32 with *lacI* ^*q*^, NruI-PvuI fragment of pABB18.5 cloned between SmaI and PvuI sites, unstable vector	
pAKB17.9	pABB32 with RA3 active partition cassette (*korA-incC-korB-orf11-parS*), EcoRV-BamHI fragment from pYC16A inserted between Ecl136II and BglII sites	
pAKB17.10	pABB35 with RA3 active partition cassette (*korA-incC-korB-orf11-parS*), EcoRV-BamHI fragment from pYC16A inserted between Ecl136II and BglII sites	
pAKB20.1	pRK415 Tc^R^ with *trfA*p-1 introduced by PCR mutagenesis with primers #18 and #19 and spontaneous deletion of 1974-bp fragment encompassing MCS, *traJ* and *oriT*	
pESB3.6	pUC18 with synthetic RA3 partition cassette (*korA-incC-korB-orf11-parS*), cloned between EcoRI and SalI sites (RA3 coordinates 5940-9800)	
**pESB30**	pABB35 with *oriT*_RK2_ *,* 218-bp fragment PCR-amplified on RK2 template using primers #13 and #14, cleaved with PscI and cloned into NcoI site, unstable, RK2 mobilizable vector	
**pESB31**	pABB35 with *oriT*_RA3,_ 166-bp fragment PCR-amplified on RA3 template using primers #15 and #16, cleaved with PscI and cloned into NcoI site, unstable, RA3 mobilizable vector	
**pESB32**	pESB30 with *klcA*p*-lacZ*, BglII-NcoI fragment from derivative of pCM132 inserted between BamHI and PscI sites, unstable, RK2 mobilizable vector	
pESB34	pESB32 with synthetic RA3 partition cassette (*korA-incC-korB-orf11-parS*), EcoRI-SalI fragment from pESB3.6 cloned between EcoRI and XhoI sites	
pJSB1.28	pJSB1.24 *Ppu*618, 618-nt PCR-amplified fragment of *P. putida* chromosome, coordinates 58074-58691	

Vectors for cloning and analysis of stabilization cassettes are in bold

Standard PCR [48] was performed with pairs of primers listed in Table 2.

**Table 2:** Oligonucleotides used in this study

No	Name	Sequence
1	*klcA*p*RA3L*	GC**GCATGC**GGGAGCGTGATCGTTACGGT
2	*klcA*p*RA3R*	GC**GGATCC**ATTGCAGCCATACGGCGAGG
3	*klcAsmaF*	GG**CCCGGG**TGCTCGTCTCGTCGGTCTG
4	*xylEPscR*	GG**ACATGT**CATCTGCACAATCTCTGCA
5	*KBRK2Apa*	CC**GAATTCGGGCCC**GAAGATGGAGATTTCCCAATGACTGC
6	*KBRK2Sal*	CC**GTCGAC**CGCTGTCTTTGGGGATCAGCCCTC
7	*lacOMCS1*	CATGGAATTGTGAGCGCTCACAATTTC**AGATCTGAATTCTACGTACTCGAG**CTCGGAATTGTGAGCGCTCACAATTTCA
8	*lacOMCS2*	CGCGTGAAATTGTGAGCGCTCACAATTCCGAG**CTCGAGTACGTAGAATTCAGATCT**GAAATTGTGAGCGCTCACAATTC
9	*parSbg2a*	GATCGGTTGCTTGTTCCACGTGGAACAAGGCCG
10	*parSbg2b*	GATCCGGCCTTGTTCCACGTGGAACAAGCAACC
11	*NrulacIF*	GG**TCGCGA**CTGAATCCGGTGAGAATGG
12	*PvulacIR*	CG**CGATCG**ATAAGCTTGCAATTCGCG
13	*oriTRK2Fs*	ACG**GTCGACACATGT**CTGGTTGGCTTGGTTTCATC
14	*oriTRK2Rs*	CG**GAATTCACATGT**TTGCCAAAGGGTTCGTGTAG
15	*oriTRA3F*	CGC**GTCGACACATGT**TTTAGCACAAGCGGCGGCAG
16	*oriTRA3R*	CG**GAATTCACATGT**AGTTAGGGGAAGCCGACGAG
17	*Nco*	A**CCATGG**TCATG
		Primers used in site-directed mutagenesis
18	mut*trfA*_*RK2*_	GTCCTTGAGAAAGGA**GACgtc** gGTTTAGCTA
19	mut*trfA*_*RK2*_	CCAATGTTTAGCTAAACc **gacGTC**TCCTTTC
20	*xhoF8180*	CGGGCCGTCGG**CTCGaG**CATCATATCGAC
21	*xhoR8180*	CGATATGATG**CtCGAG**CCGACGGCCCGC
22	*apaF9950*	CTTTCTGGTTGGCC**GgGcCC**AAAGTTTTtATCGTTTGGTTTCC
23	*apaR9950*	GAAACCAAACGATaAAAACTTT**GGgCcC**GGCCAACCAGAAAGGC
24	*Ppu618F*	CGCTGCAGAGGCCAGACCCCGTGAAATT
25	*Ppu618R*	GCAAGCTTGGTCAGCATAGTCCACCTCA
		Primers used for Real Time qPCR
26	*galKF*	ATGATCTTTCTTGCCGAGCG
27	*galKR*	AGCAGCTTTATCATCTGCCGC
28	*trfAF*	GTGAAGATCACCTACACCGGC
29	*trfAR*	TGGCAAAGCTCGTAGAACGTG
30	*repBRA3F*	CATCGAGAAGCAAAAGGCG
31	*repBRA3R*	CCAACTTGCGTAGGTCTTCCAG

Restriction enzyme recognition sites are in bold, mutated nucleotides are indicated by lowercase, *parS* palindrome is underlined

PCR-based site-directed in vitro mutagenesis was performed with mutagenic primers (Table 2) as described previously [49]. The PCR mixture after mutagenesis was treated for 1-2 h with 10 U of DpnI restriction enzyme in order to eliminate template DNA and was used for transformation of *E. coli* DH5α.

All new plasmid constructs were verified by sequencing at the DNA Sequencing and Oligonucleotide Synthesis Laboratory, Institute of Biochemistry and Biophysics, using dye terminator sequencing and an ABI 377 Perkin Elmer automated sequencer. Sequences were analyzed using Clone Manager 9.

### Plasmid construction

#### I/Construction of unstable BHR vector

Site-directed PCR mutagenesis with primers #18 and #19 introducing the T → C mutation in the -10 motif of *trfA*p together with an AatII restriction site into pRK415 was carried out to give pAKB20.1 (Fig. 1a).

pAKB20.1 was modified further by NcoI digestion and self-ligation to delete a 527-bp fragment of the *upf16.5* gene from *trfA* operon to downsize the vector and to give pABB25.

In the next step the tetracycline resistance operon (Tc^R^) of pABB25 was replaced with a shorter *cat* gene (encoding chloramphenicol acetyltransferase) conferring the Cm^R^ phenotype. The BamHI fragment with the Cm^R^ cassette from pABB705 [49], a derivative of pKRP10 [50], with NcoI and PvuII restriction sites eliminated was inserted between the BclI sites to give pABB25.1.

Site-directed mutagenesis was used to introduce a unique XhoI site downstream of *trbA* (primers #20 and #21) and an ApaI site downstream of *korA* in the *korA-incC* operon (primers #22 and #23) to give pABB27. *korA* overlaps the 5’ end of *incC1* (*incC* encodes two forms of partition protein IncC1/IncC2 with two translational starts [51]) but is translated in a different reading frame, hence the mutagenic primers #22 and #23 introduced a stop codon precluding IncC1 translation.

The new vector pABB27 was further modified by insertion of the reporter gene cassette *klcA*p-*xylE* between the *trfA* and *cat* genes to give pABB28 (Fig. 1b).

The *korB*_RK2_ gene was PCR-amplified with primers #5 and #6 on total DNA from *E. coli* DH5α(RK2) and inserted as a SalI-ApaI fragment into pABB28 digested with XhoI and ApaI to give pABB29 (Fig. 1c).

The unique NcoI and BssHII (PauI) restriction sites in pABB29 were used to introduce a new MCS sequence made from annealed oligonucleotides #7 and #8, yielding pABB32 (Fig. 1d).

*lacI*^*q*^ was amplified on pGBT30 [34] using primers #11 and #12 and inserted as NruI-PvuI fragment into pABB32 between SmaI and PvuI sites to obtain the final pABB35 vector (Fig. 1e).

PCR-amplified 218-bp *oriT*_RK2_ on RK2 template (primers #13 and #14) and 166-bp *oriT*_RA3_ PCR-amplified on RA3 template (primers #15 and #16), were inserted as PscI fragments into the unique NcoI site of pABB35 to give mobilizable vectors pESB30 and pESB31, respectively.

#### II/Construction of *klcA*p_RA3_-*xylE* and *klcA*p_RA3_-*lacZ* reporter cassettes

Two reporter genes, *xylE* of pWWO from *P. putida* encoding catechol 2,3-dioxygenase [10], and promoter-less *lacZ* from pCM132 coding for β-galactosidase [52], were cloned into appropriate vectors.

The strong promoter *klcA*p [40] was PCR-amplified on RA3 template using primers #1 and #2 and cloned as an SphI-BamHI fragment into pPT0I vector [10] upstream of the *xyl* operon to yield pABB18.1. Subsequently, pABB18.1 was cut with HpaI and NcoI, filled in with PolI Klenow fragment and self-ligated to remove a 561-bp fragment upstream of the *xylE* gene (pABB18.2). *klcA*p*-xylE* was PCR-amplified from pABB18.2 (primers #3 and #4) and cloned as a SmaI-PscI fragment into pABB19 [49] to provide a cassette with a bi-directional Rho-independent transcription terminator, giving pABB18.3. The role of the transcription terminator inserted at the end of the reporter gene cassette was to prevent transcriptional spillover from the strong *klcA* promoter and to protect the *klcA*p-*xylE* cassette against transcription coming from inserts in the constructed vectors, as a new MCS was planned to be cloned next to the NcoI site. The SmaI-NcoI fragment encoding *klcA*p_RA3_-*xylE-*T_*pro/lyz*P1_ was inserted into pABB27 to give pABB28.

pCM132 [52] carrying promoter-less *lacZ* orf was cut with SphI and ligated with self-annealed oligonucleotide #17 to remove the SphI and insert an NcoI restriction site downstream of *lacZ* (pCM132Nco). The BglII-NcoI fragment from pCM132Nco carrying a promoter-less *lacZ* cassette was inserted into pESB30 between the BamHI and PscI sites to replace *xylE* and transcriptionally fuse *lacZ* to the strong *klcA* promoter and to construct pESB32 (Fig. 1f).

#### III/Cloning the partitioning cassettes into the test vectors

Chromosomal *parA-parB* operon of *P. aeruginosa* had been cloned earlier under the control of *tac*p and *lacI*^*q*^ in pKLB3 [29]. The DraI-SalI fragment of pKLB3 bearing *lacI*^*q*^*-tac*p*-parA-parB* was re-cloned between the SnaBI and XhoI sites of pABB32 (Fig. 1d), yielding pABB33. A centromere-like sequence *parS*_*2/3*_ [29] made from annealed oligonucleotides #9 and #10 (Table 2) was cloned into BglII-cut pABB33 to give pABB34 with the complete stabilization cassette from *P. aeruginosa*.

The EcoRV-BamHI fragment of pYC16A carrying the active partition cassette *korA-incC-korB-orf11-parS* from RA3 plasmid [40, 41] was cloned into pABB32 and pABB35 vectors digested with Ecl136II and BglII to obtain pAKB17.9 and pAKB17.10, respectively. In the case of pESB34, the EcoRI-SalI fragment from pESB3.6 carrying *korA-incC-korB-orf11-parS* of RA3 was cloned between the EcoRI and XhoI sites of pESB32.

#### IV/Construction of *P. putida* KT2440 *tra*_RA3_*korC*_RA3_ Km^R^ helper strain

pJSB1.28 is a high copy number plasmid based on pMB1 replicon unable to replicate in *P. putida.* It is derivative of pJSB1.24 that carries the conjugative transfer module of plasmid RA3 (RA3 coordinates 9437- 33857) together with the *korC* gene (RA3 coordinates 3093-3705) encoding an indispensable transcriptional repressor [49]. A short region of *P. putida* KT2440 chromosome (coordinates 58074-58691) was PCR-amplified with the use of primers # 24 and #25 (Table 2) and cloned between PstI and HindIII sites to facilitate integration of pJSB1.28 into the chromosome by homologous recombination. DH5α (pJSB28) donor strain was conjugated with *P. putida* KT2440 and integrants were selected on M9 plates [47] with 0.1 % glucose and kanamycin (50 μg ml^−1^) without thiamine to eliminate DH5α that requires thiamine to grow on minimal medium. The integration of pJSB1.28 into the chromosome of KT2440 *tra*_RA3_*korC*_RA3_ Km^R^ was verified by PCR.

### Transformation and conjugation procedures

Competent *E. coli* and *P. putida* cells were prepared by the standard CaCl_2_ method [47]. Competent cells of *P. aeruginosa* were prepared according to the method of Irani and Rowe [53]. *E. coli* S17-1 strain was transformed with test plasmids carrying *oriT*_RK2_ and such transformants were used as donors in conjugation with recipient *A. tumefaciens* LBA1010R Rif^R^ strain. Overnight cultures of the donor and recipient strains (100 μl each) were mixed on L agar plate and incubated overnight at 28 °C. Bacteria from the plate were suspended in L broth and serial dilutions of the suspension were plated on L agar with rifampicin (150 μg ml^−1^) and chloramphenicol (50 μg ml^−1^) and incubated at 28 °C. Alternatively, *P. putida* KT2440 *tra*_RA3_-*korC* Km^R^ strain was transformed with pABB35 derivatives carrying *oriT*_RA3_ (e.g., pESB31) and such transformants were used as donor strains.

### Standard plate test of plasmid stability

Cultures of hosts carrying various plasmids were grown in L broth with selective antibiotics at 37 °C or 28 °C. Plasmid content in the initial cultures was assessed by plating 100 μl of diluted cultures onto L agar to get approximately 100–200 colonies (usually 10^6^-fold dilution) and then re-streaking 100 colonies onto L agar with the selective antibiotic. Plasmid retention was expressed as the percentage of Cm^R^ colonies. The cultures were grown in L broth without antibiotics for up to 60 generations (diluted 10^5^-fold at the start and every 20 generations) and plasmid retention was analyzed as above. No IPTG (isopropyl β-D-1-thiogalactopyranoside) was added to the cultures with the exception of experiments with *E. coli* DH5α (pABB34) where the effect of IPTG concentration was analyzed.

### Rapid plate screening for plasmid retention

Transformants were grown overnight and diluted as described above. Every 20 generations 100 μl of 10^6^-fold diluted culture was plated on L agar (for plasmids with *klcA*p*-xylE* fusion) or L agar with X-gal (for plasmids with *klcA*p*-lacZ* fusion). Colonies formed by bacteria with plasmids carrying the *klcA*p*-xylE* fusion become yellow after being sprayed with 10 mM catechol solution. Plasmid retention was calculated as the percentage of yellow colonies or blue colonies on X-gal for plasmids with *klcA*p*-lacZ* fusion.

### Catechol 2,3-dioxygenase activity assay

The level of *xylE* expression from *klcA*p was determined by an enzymatic assay in extracts from logarithmically growing cultures of *E. coli* DH5α transformants using a standard method [27]. Protein concentration was assayed by the Bradford method [54]. One unit of catechol 2,3-dioxygenase activity is defined as the amount of enzyme necessary to convert 1 μmol of catechol to the yellow hydroxymuconic semialdehyde in 1 min under standard conditions.

Simplified XylE activity assay was applied to estimate plasmid stability in liquid cultures. Overnight cultures of *E. coli* DH5α transformants carrying test plasmids with *klcA*p*-xylE* (grown without selection for 20 or 40 generations) were diluted 100-fold, the cell densities were estimated by OD_600_, 1/10 volume of 10 mM catechol was added and absorbance at 405 nm was measured after 5 min. The OD_405_/OD_600_ ratio clearly differentiated strains with plasmids of various stability. To quantify plasmid retention a high-throughput procedure with 100-well plates (Labsystems Honeycomb 2 plate) and a Bioscreen C Microbiology Reader Analyser (Labsystems) was used. Details are described in the Results section.

### Determination of plasmid copy number by quantitative real-time PCR (qPCR)

The copy number of pRK415 and its derivatives was measured by qPCR using the SYBR® Green JumpStart™ Taq ReadyMix kit (Sigma). The single-copy *galK*, gene from *E. coli* chromosome, was used as the chromosomal reference gene (primers #26 and #27) for all strains. The *trfA* gene of RK2 was used as the plasmid reference gene (primers #28 and #29) for pRK415 derivatives and pMPB9.90 (*araBADp-trfA*_RK2_) whereas *repB* gene was amplified for plasmid RA3 (primers #30 and #31) (Table 2). Total DNA was purified from 4 ml of stationary-phase cultures using Genomic Mini purification kit (A&A Biotechnology), treated with an appropriate restriction enzyme to linearize the plasmid DNA and to fragment chromosomal DNA and then used as a template in qPCR. Plasmid copy number (PCN) was calculated relatively to the chromosomal marker on the basis of at least three biological replicates with three technical replicates per strain and average results with standard deviation are reported. The amplification, detection and analysis were carried out in the Laboratory of Genetic Modification Analyses of IBB PAS on an Applied Biosystems 7500 apparatus.
